# Retraction: Histological Patterns of Mycophenolate Mofetil-Induced Gastrointestinal Mucosal Injury in Renal Transplant Recipients

**DOI:** 10.7759/cureus.r235

**Published:** 2026-07-17

**Authors:** Nandita Chaudhary, Sumit Sachan, Abdullah Ansari, Neha Nigam, Ram N Rao, Anupma Kaul

**Affiliations:** 1 Pathology, Sanjay Gandhi Postgraduate Institute of Medical Sciences, Lucknow, IND; 2 Anaesthesiology, Sanjay Gandhi Postgraduate Institute of Medical Sciences, Lucknow, IND; 3 Nephrology, Institute of Medical Sciences, Banaras Hindu University, Varanasi, IND; 4 Nephrology, Sanjay Gandhi Postgraduate Institute of Medical Sciences, Lucknow, IND

This article has been retracted by the Editor-in-Chief at the request of the authors due to an unresolved dispute regarding authorship.

Following publication, a concern was raised regarding the accuracy of the author list. The editorial team requested that the parties involved work together to resolve the matter and, where appropriate, provide documentation of each author's contribution in line with ICMJE authorship criteria.

The parties were unable to reach an agreement on the appropriate authorship of this article. As the journal cannot be assured that the published author list accurately reflects those who meet ICMJE criteria for authorship, and given the inability to resolve this dispute despite reasonable opportunity to do so, the journal has determined that retraction is necessary to preserve the integrity of the scholarly record.

